# A Comprehensive Strategy to Discover Inhibitors of the Translesion Synthesis DNA Polymerase κ

**DOI:** 10.1371/journal.pone.0045032

**Published:** 2012-10-08

**Authors:** Kinrin Yamanaka, Dorjbal Dorjsuren, Robert L. Eoff, Martin Egli, David J. Maloney, Ajit Jadhav, Anton Simeonov, R. Stephen Lloyd

**Affiliations:** 1 Department of Physiology and Pharmacology, Oregon Health & Science University, Portland, Oregon, United States of America; 2 Center for Research on Occupational and Environmental Toxicology, Oregon Health & Science University, Portland, Oregon, United States of America; 3 Department of Molecular and Medical Genetics, Oregon Health & Science University, Portland, Oregon, United States of America; 4 National Center for Advancing Translational Sciences, National Institutes of Health, Bethesda, Maryland, United States of America; 5 Department of Biochemistry and Molecular Biology, University of Arkansas for Medical Sciences, Little Rock, Arkansas, United States of America; 6 Department of Biochemistry, Center in Molecular Toxicology and Vanderbilt Institute of Chemical Biology, Vanderbilt University School of Medicine, Nashville, Tennessee, United States of America; Louisiana State University and A & M College, United States of America

## Abstract

Human DNA polymerase kappa (pol κ) is a translesion synthesis (TLS) polymerase that catalyzes TLS past various minor groove lesions including *N* ^2^-dG linked acrolein- and polycyclic aromatic hydrocarbon-derived adducts, as well as *N* ^2^-dG DNA–DNA interstrand cross-links introduced by the chemotherapeutic agent mitomycin C. It also processes ultraviolet light-induced DNA lesions. Since pol κ TLS activity can reduce the cellular toxicity of chemotherapeutic agents and since gliomas overexpress pol κ, small molecule library screens targeting pol κ were conducted to initiate the first step in the development of new adjunct cancer therapeutics. A high-throughput, fluorescence-based DNA strand displacement assay was utilized to screen ∼16,000 bioactive compounds, and the 60 top hits were validated by primer extension assays using non-damaged DNAs. Candesartan cilexetil, manoalide, and MK-886 were selected as proof-of-principle compounds and further characterized for their specificity toward pol κ by primer extension assays using DNAs containing a site-specific acrolein-derived, ring-opened reduced form of γ-HOPdG. Furthermore, candesartan cilexetil could enhance ultraviolet light-induced cytotoxicity in xeroderma pigmentosum variant cells, suggesting its inhibitory effect against intracellular pol κ. In summary, this investigation represents the first high-throughput screening designed to identify inhibitors of pol κ, with the characterization of biochemical and biologically relevant endpoints as a consequence of pol κ inhibition. These approaches lay the foundation for the future discovery of compounds that can be applied to combination chemotherapy.

## Introduction

Cells employ multiple mechanisms to repair or tolerate DNA lesions in order to maintain genomic integrity. Translesion DNA synthesis (TLS) is one of the mechanisms used to tolerate unrepaired DNA lesions [1]–[6]. DNA polymerase κ (pol κ) is a TLS polymerase that has been shown to catalyze TLS past a variety of DNA lesions, being particularly proficient in the bypass of minor groove *N* ^2^-dG lesions, including the acrolein-derived adducts γ-HOPdG and its ring-opened reduced form, DNA–peptide cross-links, and DNA–DNA interstrand cross-links (ICLs), as well as adducts induced by activated polycyclic aromatic hydrocarbons such as benzo[*a*]pyrene diolepoxide [7]–[14]. Importantly, pol κ has been demonstrated to be involved in the tolerance of ICLs induced by a chemotherapeutic agent, mitomycin C [12]. In addition to its role in the bypass of *N* ^2^-dG lesions, pol κ has also been shown to play a role in the processing of various ultraviolet (UV) light-induced DNA lesions [15]–[17].

Many clinically relevant chemotherapeutic agents, including mitomycin C, cisplatin, and nitrogen mustard, target tumor cells by virtue of their ability to covalently cross-link complementary DNA strands, introducing ICLs into the genome. These ICL-inducing agents are powerful chemotherapeutic agents as the ICL interferes with vital cellular processes such as DNA replication, RNA transcription, and recombination by preventing transient DNA strand separation [18]–[21]. Therefore, although TLS is an essential process for cells to survive genotoxic stress, the ability of pol κ to bypass ICLs could limit the efficacy of these agents. Critical to this point are data demonstrating that the effectiveness of mitomycin C was increased when pol κ expression was suppressed by siRNA [12].

Germane to these observations, previous reports have suggested that pol κ may play a role in glioma development and therefore serve as a potential target for novel routes of therapies. Gliomas are the most common form of primary brain cancer and represent what is currently a generally incurable tumor in humans. These tumors are highly resistant to current treatment strategies, including chemotherapy with alkylating agents such as temozolomide, leading to median survival of patients with high-grade gliomas of only 1 year post diagnosis [22]. Therefore, there is an urgent need for development of new therapies. Significantly, the level of pol κ has been shown to be upregulated in tumors from glioma patients, with its level being highly correlated with the grades of disease. Moreover, glioma patients expressing high levels of pol κ have an even poorer prognosis [23]. Collectively, these data suggest a potential role for pol κ in the development of glioma. Thus, the identification of small molecule inhibitors targeting pol κ may be crucial for improving the therapeutic efficacy of chemotherapeutic agents.

To the best of our knowledge, only one selective small molecule inhibitor of pol κ has been identified to date: a natural product, 3-*O*-methylfunicone [24]. This compound exhibits selectivity against Y-family DNA polymerases, and importantly, among the Y-family polymerases investigated, it shows the highest potency towards pol κ at IC_50_ of 12.5 µM. However, the utilization of 3-*O*-methylfunicone for therapeutic purposes is limited by its low potency. Additionally, given a lack of analogues and structure-activity relationship of this compound, it is unclear whether 3-*O*-methylfunicone-based agents can be developed into efficient therapeutics. Thus, in search for compounds with improved potency, a quantitative high-throughput screening (qHTS) of libraries of bioactive molecules composed of 15,805 members was carried out. Here we report the new strategies to identify small molecule inhibitors of pol κ.

## Materials and Methods

### Materials

1 M Tris-HCl was purchased from Invitrogen (Grand Island, NY), while Tween-20, KCl, MgCl_2_, and dithiothreitol (DTT) were purchased from Sigma–Aldrich (St. Louis, MO). Black 384-well and 1,536-well plates were purchased from Greiner Bio-One (Monroe, NC). [γ-^32^P]ATP was obtained from PerkinElmer Life Sciences (Waltham, MA). P-6 Bio-Spin columns were obtained from Bio-Rad (Hercules, CA). T4 polynucleotide kinase and 100 bp DNA ladder were purchased from New England BioLabs (Beverly, MA). Human pol κ (residues 19–526) was purified as previously reported [25]. Human pol η (residues1–437) was purified following the same procedures as the purification of pol κ. Human pol ι was purchased from Enzymax Inc. (Lexington, KY). Yeast pol δ was a generous gift from Dr. Peter M. J. Burgers (Washington University, St. Louis, MO). Dimethyl sulfoxide (DMSO, certified ACS grade), paraformaldehyde, glacial acetic acid, crystal violet, and ethidium bromide were purchased from Fisher Scientific (Pittsburgh, PA). Clear 96-well plates and white/clear bottom 96-well plates were purchased from BD Falcon (Franklin Lakes, NJ) and Corning (Corning, NY), respectively. CellTiter-Glo Luminescent Cell Viability Assay was obtained from Promega (Madison, WI). Xeroderma pigmentosum variant (XP-V), XP30RO cells were generously supplied by Dr. James E. Cleaver (University of California, San Francisco, San Francisco, CA) and were generated as described previously [26].

### Oligodeoxynucleotides synthesis

The three oligodeoxynucleotides used in the qHTS (see below under *qHTS*) were purchased from Biosearch Technologies, Inc., (Novato, CA). Control unadducted oligodeoxynucleotides and an oligodeoxynucleotide adducted with acrolein-derived ring-opened reduced form of γ-HOPdG were the generous gifts of Dr. Carmelo J. Rizzo (Vanderbilt University, Nashville, TN).

### qHTS

#### Fluorogenic substrate

The substrate used in qHTS was made by the annealing of unlabeled oligodeoxynucleotide primer (5′-TCACCCTCGTACGACTC-3′) and TAMRA-labeled reporter strand (5′-TTTTTTTTTTGC-6-TAMRA-3′) to oligodeoxynucleotide template labeled with BHQ-2 (5′-BHQ-2-GCAAAAAAAAAAGAGTCGTACGAGGGTGA-3′) as originally described by Dorjsuren *et al.* [27]. The annealing mixture of unlabeled primer, TAMRA-labeled reporter, and BHQ-2-labeled template in buffer containing 50 mM Tris-HCl, pH 8.0, 100 mM NaCl, and 5 mM MgCl_2_ was heated at 95°C for 5 min and allowed to cool gradually to room temperature. The substrate was then stored at −20°C as 50 µM stock.

#### Compound libraries

The screening collection of 15,805 members included the following libraries with the number of compounds indicated in parentheses: NCGC Pharmaceutical Collection (NPC) [28], MicroSource Spectrum collection (2,031), TimTec natural products (400), the LOPAC^1280^ collection from Sigma–Aldrich (1280), Tocris (1624), Prestwick (1597), BioMol (1943), Pharmacopeia (1648), and NCGC chemistry analogues and several additional small-size collections of bioactives.

#### qHTS assay protocol

qHTS was performed in 50 mM Tris-HCI, pH 8.0, 40 mM NaCl, 1 mM MgCl_2_, 0.01% Tween-20, 2 mM DTT, and 100 µM dTTP. 3 µL of reagents (buffer as negative control and pol κ in the remaining plate at 10 nM final concentration) were dispensed by Flying Reagent Dispenser™ (FRD) (Beckman Coulter, Inc., Fullerton, CA) into a 1,536-well plate. Compounds were delivered as 23 nL in DMSO solution via pintool transfer; vehicle-only control consisted of 23 nL DMSO. The plate was incubated for 15 min at room temperature, and then 1 µL of substrate (50 nM final concentration) was added to initiate the reaction. The plate was immediately transferred into ViewLux reader for kinetic fluorescence data collection.

### Radioactive gel-based primer extension assays

The reactions with pol κ, pol η, and pol ι were carried out in the buffers containing 25 mM Tris-HCl, pH 7.5, 8 mM MgCl_2_, 10% glycerol, 100 µg/mL bovine serum albumin, and 5 mM DTT. Buffers for pol δ reactions contained 40 mM Hepes-KOH, pH 6.8, 10% glycerol, 200 µg/mL bovine serum albumin, 1 mM DTT, and 8 mM MgCl_2_. Final concentration of pol κ, pol η, pol ι, or pol δ in the reaction was 0.25 nM, 1 nM, 2.5 nM, or 4 nM, respectively. In this assay, a polymerase was preincubated with the compound for 15 min at room temperature. Primer extensions were then initiated by the addition of DNA substrate at final concentration of 5 nM and carried out for 30 min at room temperature in the presence of 100 µM dCTP and dGTP. The reactions were terminated by the addition of an equal volume of a dye solution containing 95% (v/v) formamide, 10 mM EDTA, 0.03% (w/v) xylene cyanol, and 0.03% (w/v) bromphenol blue. Reaction products were separated through a 15% acrylamide gel containing 8 M urea and visualized with a PhosphorImager screen. The ratio of extended primer to the total amount of primer was measured using ImageQuant 5.2 software to calculate the percentage of primer extended. IC_50_ values were determined by fitting the data to variable slope four-parameter equations using GraphPad Prism 5.

### DNA intercalation assay

Candesartan cilexetil or ethidium bromide was incubated with 1.5 nmol of double-stranded DNA ladder for 15 min at room temperature in the buffer containing 50 mM Tris-HCl, pH 8.0, 0.01% Tween-20, 1 mM DTT, and 40 mM NaCl. The reaction mixtures were run on 1% agarose gel and stained with ethidium bromide (0.5 µg/ml) for visualization. The amounts of candesartan cilexetil or ethidium bromide used for the reactions were indicated in figure legends.

### Cell survival assays

Cell survival assays were carried out using crystal violet assays and CellTiter-Glo Luminescent Cell Viability Assays. In crystal violet assays, XP30RO cells were plated into clear 96-well plates at a density of 8000 cells/mL and incubated overnight at 37°C, 5% CO_2_. The cells were treated with (1) inhibitor alone for 6 h or (2) UV alone or (3) inhibitor alone for 6 h followed by UV irradiation. After 2 days, cells were fixed with 4% paraformaldehyde and stained with 0.5% crystal violet dye. The dye was dissolved in 10% acetic acid and absorbance measurement was taken at OD_595_ nm with Infinite® M200 plate reader (Tecan, Durham, NC). For the CellTiter-Glo Luminescent Cell Viability Assays, the same procedures as crystal violet assays were used except that white/clear bottom 96-well plates were used and cell viability was measured following manufacturer's recommendations. The concentration of candesartan cilexetil and the dose of UV were indicated in figure legends.

## Results

### qHTS

**Figure 1:**
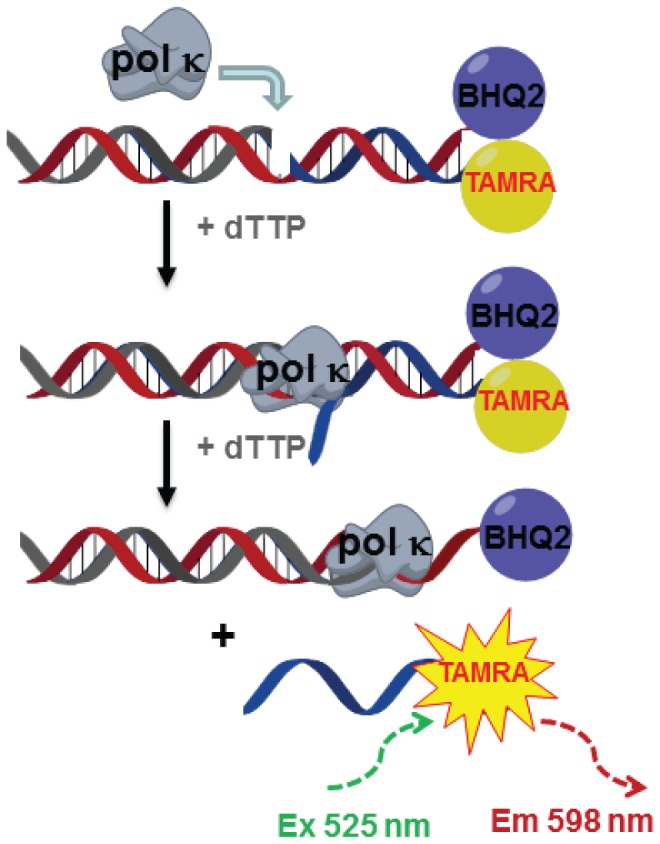
Principle of the fluorescence-based polymerase-catalyzed strand displacement assay. Pol κ-catalyzed DNA synthesis displaces TAMRA-labeled reporter strand. This process relieves the quenching effect of BHQ-2, leading to an increase in fluorescence signals.

**Figure 2:**
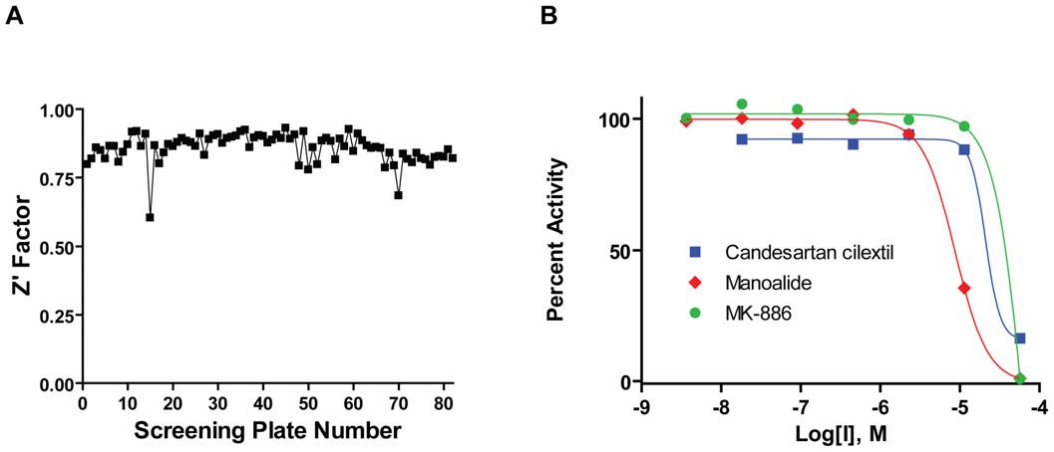
Summary of results from qHTS. (**A**) Excellent Z′ factor of >0.75 was maintained throughout the screen. (**B**) Concentration-response curves of top hits derived from the screen.

To lay the foundation for the methodologies to discover and characterize inhibitors of pol κ, we used a fluorescent reporter strand displacement assay (Figure 1 and [27]). The library of bioactive small molecules was tested as seven-point dilution series, with the final concentrations ranging from 2.9 nM to 57 µM. The statistical performance of the screening assay as expressed by its Z′ factor remained consistently high with only few deviations, at an average of 0.85 (the maximum Z′ factor possible is 1.0, while values of greater than 0.5 are considered an indication of a highly stable screening assay) (Figure 2A).

The concentration-response screen paradigm used here [29] allowed for calculation of an inhibitory potency IC_50_ value for each positive hit in the primary screen, as well as an evaluation of the shape and other characteristics of the concentration response curve. A total of 60 hits displaying full concentration-response curves and IC_50_ values of less than 50 µM were identified (the qHTS results are available in PubChem under Assay Identifier 588579, http://pubchem.ncbi.nlm.nih.gov/ and Table S1), and concentration response curves of representative top hits are shown in Figure 2B. The results from these experiments were combined with the radioactive gel-based secondary primer extension assays described below in order to nominate a small subset of hits for detailed characterization.

### Candesartan cilexetil, manoalide, and MK-886 inhibit pol κ-catalyzed primer extensions on non-damaged DNA in vitro

**Figure 3:**
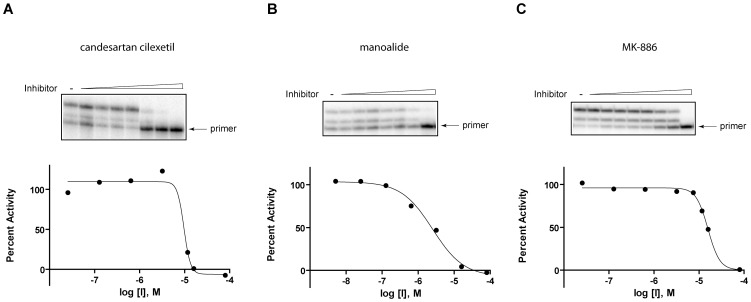
Dose-response activities of (A) candesartan cilexetil, (B) manoalide, and (C) MK-886 on non-damaged DNAs. Representative data from three independent experiments are shown. The gels shown on the top panel were used to generate dose-response curves shown on the bottom panel.

**Figure 4.Summary:**
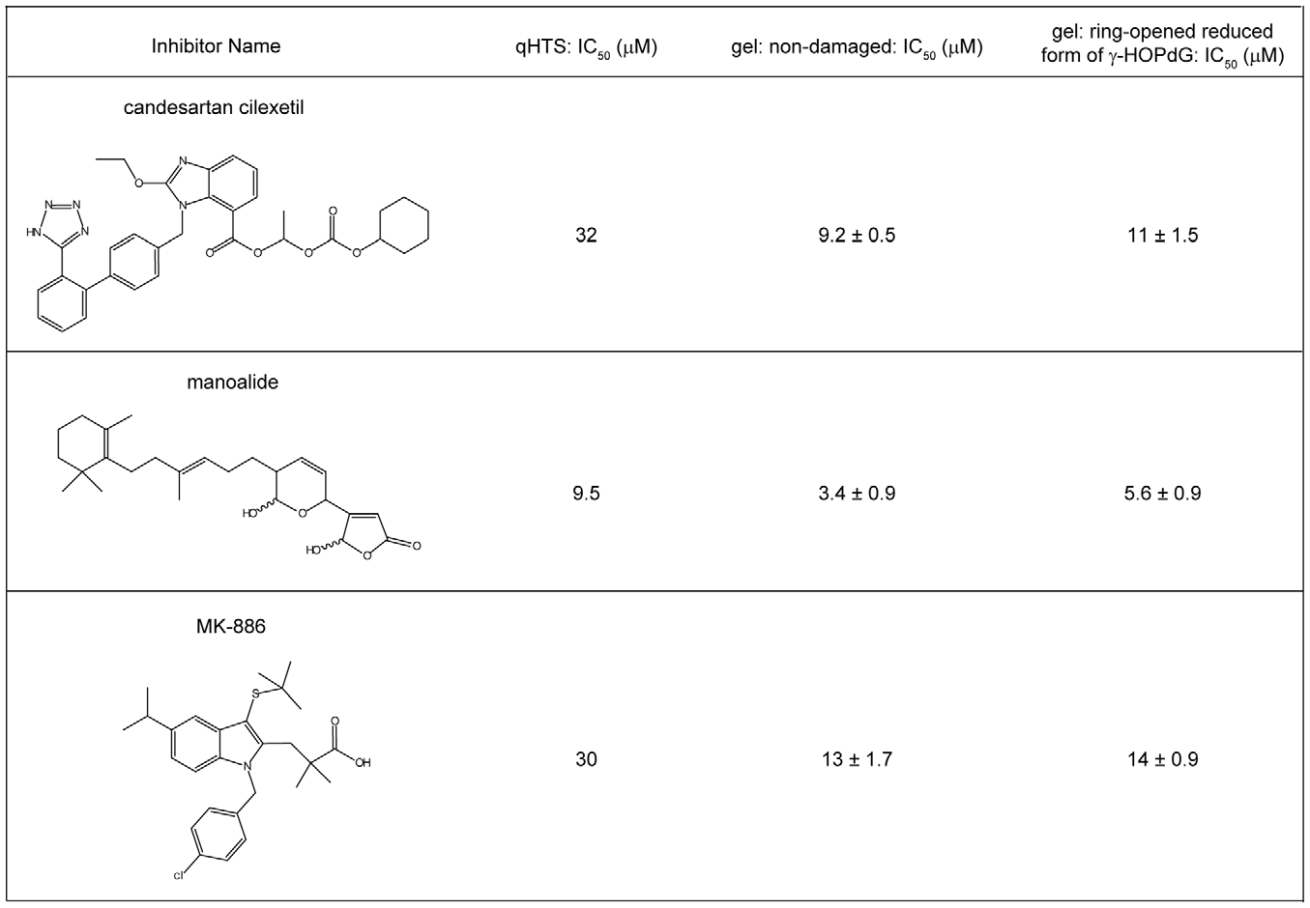
of IC_50_s of candesartan cilexetil, manoalide, and MK-886 from qHTS and primer extension assays. In primer extension assays, three independent experiments were carried out, and IC_50_s are shown as average ± standard error.

In order to confirm the reliability of the qHTS and to serve as a proof-of-principal that this method can be utilized in the further future screenings to identify pol κ inhibitors with potential for drug development, 60 of the hits that were identified through qHTS were analyzed by an orthogonal detection method, consisting of a radioactive gel-based primer extension assays using non-damaged DNA. Initially, the assay was carried out at 80 µM of each compound in order to identify false-positive compounds that were inactive against pol κ, even at this high concentration (Table S1). Using this assay, 3 compounds were shown to have minimal effect on pol κ and thus were not considered in further analyses. Additionally, 5 compounds interfered with the migration of the DNA into the gel and were excluded from further analyses due to potential solubility problems and a lack of availability of these compounds in significant amounts. Thus, a total of 8 compounds were excluded from further analyses. The remaining 52 compounds showed a range of inhibitory activity against pol κ at 80 µM. Based on the compounds' activity in the primer extension assays, the presence of reactive functional group(s) in the compounds, their tendency to appear as actives in a large number of internally-conducted screens (i.e., their promiscuity), and the commercial availability of the compounds to enable further studies, candesartan cilexetil, manoalide, and MK-886 were selected as compounds that would serve as proof-of-principal chemicals for further biochemical and biological assay development. The IC_50_ values of these compounds were examined by primer extension assays with various concentrations of the compounds, with the results showing that these compounds inhibited pol κ activity in a dose-dependent manner, and the IC_50_s of candesartan cilexetil, manoalide, and MK-886 on non-damaged DNA were 9.2±0.5 µM, 3.4±0.9 µM, and 13±1.7 µM, respectively (Figure 3 and Figure 4). Despite significant differences between the fluorescence substrate-based HTS method and the radioactive gel-based primer extension assay, IC_50_s obtained from qHTS and primer extension assays were found to be well-correlated (Figure 4).

### Candesartan cilexetil, manoalide, and MK-886 inhibit pol κ-catalyzed TLS past acrolein-derived ring-opened reduced form of γ-HOPdG lesions in vitro

**Figure 5:**
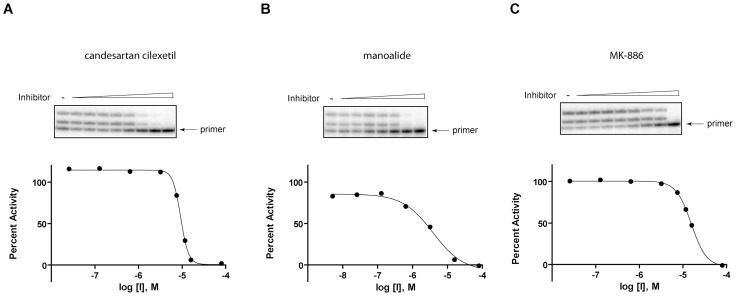
Dose-response activities of (A) candesartan cilexetil, (B) manoalide, and (C) MK-886 on adducted DNAs. Representative data from three independent experiments are shown. The gels shown on the top panel were used to generate dose-response curves shown on the bottom panel.

The primary role of pol κ is to bypass DNA lesions. Therefore, it was critical to establish the assays to investigate the capability of these compounds to inhibit lesion bypass activity of pol κ. Radioactive gel-based primer extension reactions were carried out using a DNA substrate containing a site-specific acrolein-derived ring-opened reduced form of γ-HOPdG, a lesion that pol κ can efficiently bypass *in vitro* [13]. As shown in Figure 5, all these compounds were capable of inhibiting pol κ-catalyzed TLS past the lesion in a dose-dependent manner with potencies similar to those shown for the inhibition of pol κ-catalyzed primer extensions on non-damaged DNA. The IC_50_s obtained from these reactions were 11±1.5 µM for candesartan cilexetil, 5.6±0.9 µM for manoalide, and 14±0.9 µM for MK-886 (Figure 4).

### Candesartan cilexetil enhances UV-induced cellular toxicity

**Figure 6:**
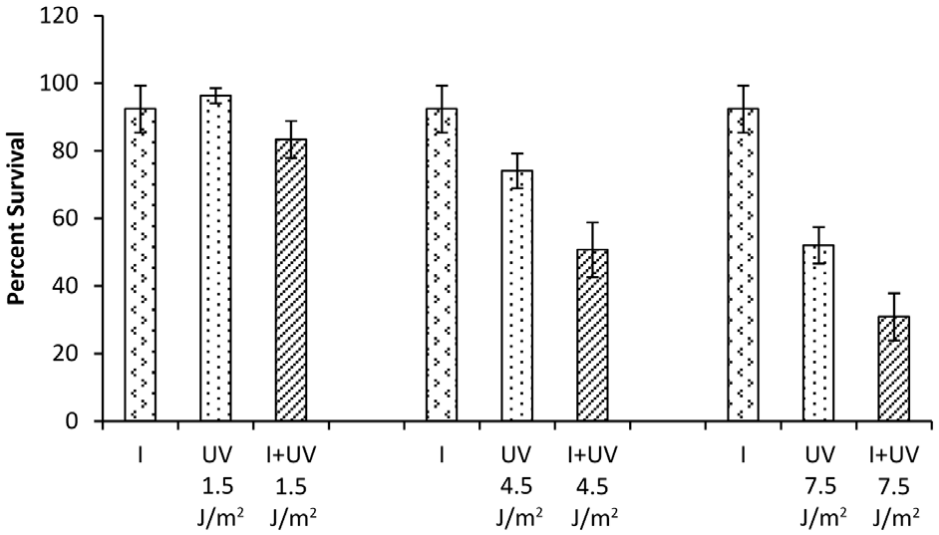
Effect of candesartan cilexetil on UV-induced cytotoxicity using crystal violet assay. XP30RO cells were treated with 24 µM of candesartan cilexetil alone, UV alone at 1.5 J/m^2^, 4.5 J/m^2^, or 7.5 J/m^2^, or in combination. Percent survival was calculated by normalizing the data using the cell survival of untreated cells. The data were obtained from three independent experiments. Error bars represent standard deviations. I: candesartan cilexetil.

To assess the potential biological efficacy that would be conferred by inhibiting pol κ, it is necessary to maximize the difference in the cellular endpoint between wild type and pol κ-deficient cells. In order to address this goal, we chose to exploit differential cytotoxicity as an endpoint. Even though the effect of reduction in cellular pol κ on mitomycin C-induced cellular toxicity has been shown, this enhanced cytotoxicity was only slightly less than 2-fold [12]. However, Ziv *et al.* previously reported that XP-V cells had 3.5- to 5-fold enhanced sensitivity to UV irradiation when pol κ was depleted versus control cells [16]. Thus, to demonstrate that at least a subset of these small molecule inhibitors could exhibit biological efficacy in a cytotoxicity assay, we hypothesized that a pol κ inhibitor should be capable of enhancing the cytotoxic effect of UV exposure in XP-V cells. To test this hypothesis, XP-V cells were treated with candesartan cilexetil or UV alone, or in combination, and assayed for cell viability using a crystal violet staining assay. As shown in Figure 6, the inhibitor potentiated the cytotoxicity of UV at higher UV doses (4.5 and 7.5 J/m^2^). The assay was also repeated with the CellTiter-Glo Luminescent Cell Viability Assay, that measures ATP content of the cells, and comparable results were obtained (Figure S1). These data suggest that this assay may be a useful tool in the design of a cell-based high throughput screening assay for the identification and characterization of additional compounds and that candesartan cilexetil may inhibit intracellular pol κ. Cell viability was also investigated with manoalide and MK-886. Although both compounds could inhibit pol κ activity *in vitro*, they failed to enhance UV toxicity in these cells under the conditions investigated (data not shown).

### Polymerase selectivity of candesartan cilexetil to inhibit replicative and other TLS polymerase-catalyzed primer extensions on non-damaged DNA in vitro

In order to address the specificity of candesartan cilexetil in inhibiting DNA polymerases, radioactive gel-based primer extension assays were carried out on non-damaged DNA with other TLS polymerases (pol η and pol ι) (Table 1). These data revealed that candesartan cilexetil inhibited both of these polymerases at concentrations comparable to that of pol κ, suggesting that this compound may serve as a general TLS polymerase inhibitor and possibly that it could simply be a general polymerase inhibitor. However, the cellular data using the XP-V cells suggested that this compound is unlikely to be an inhibitor of any of the replicative polymerases, since the cell survival was not altered in the presence of 24 µM candesartan cilexetil. In order to address this assumption, primer extension reactions were carried out with yeast pol δ with several concentrations of candesartan cilexetil, with the maximum concentration being 80 µM. These data revealed that only at 80 µM concentration was there detectable inhibition of DNA synthesis (data not shown) and since all lower concentrations tested showed minimal inhibition, IC_50_ values could not be determined. These data are consistent with the normal cell growth observed in tissue culture. Thus, we conclude that candesartan cilexetil is not a general polymerase inhibitor, but is not selective toward pol κ.

**Table 1:** Summary of IC_50_s of candesartan cilexetil for the inhibition of pol η and pol ι in replicating non-damaged DNAs.

IC_50_ (µM)	
pol η	pol ι
11.2±1.0	6.2±0.6

IC_50_s were obtained from three independent experiments and shown as average ± standard error.

### Candesartan cilexetil does not intercalate into DNA

**Figure 7:**
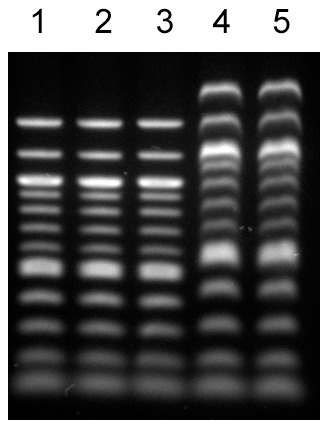
The non-DNA-intercalating property of candesartan cilexetil. Candesartan cilexetil or ethidium bromide was incubated with double-stranded DNA ladder, and the reaction products were run on 1% agarose gel and stained with ethidium bromide. Lane 1: No compound. Lane 2: 0.75 nmol of candesartan cilexetil was incubated with 1.5 nmol DNA. Lane 3: 3 nmol of candesartan cilexetil was incubated with 1.5 nmol DNA. Lane 4: 0.75 nmol of ethidium bromide was incubated with 1.5 nmol DNA. Lane 5: 3 nmol of ethidium bromide was incubated with 1.5 nmol DNA.

It is possible that candesartan cilexetil inhibited pol κ activity by blocking the access of pol κ to DNA. Thus, the property of this compound to intercalate into DNA was investigated. As shown in Figure 7, upon mixing of candesartan cilexetil or a control well-known DNA intercalator, ethidium bromide, with double-stranded DNA, the bands shifted upwards in the presence of ethidium bromide, while no difference in DNA migration pattern was observed with candesartan cilexetil compared to control. These results suggest that candesartan cilexetil is unlikely to intercalate into DNA.

## Discussion

Despite the potential significance of targeting pol κ in cancer therapeutics, limited investigations have been reported concerning the discovery of pol κ inhibitors. Although compounds with inhibitory activity against pol κ have been identified, most of these inhibitors were discovered as part of screens of natural products against any DNA polymerase and their therapeutic potential may be limited primarily due to either low potency or poor selectivity. For example, a derivative of a natural product kohamaic acid, (1S*,4aS*,8aS*)-17-(1,4,4a,5,6,7,8,8a-octahydro-2,5,5,8a-tetramethylnaphthalen-1-yl)heptadecanoic acid and a derivative of vitamin K_2_ and vitamin K_3_, MK-2, have been shown to inhibit pol κ activity with IC_50_s of 7.2 µM and 35.3 µM, respectively [30], [31]. Pol κ activity is also inhibited by C12:0-Acyl juglone and C18:1-Acyl juglone with IC_50_s of 6.8 µM and 8.1 µM, respectively [32]. Glycyrrhetinic acid is another compound that inhibits pol κ activity (IC_50_ of 15.8 µM) [33]. Penicilliols A and B are other natural products that inhibit pol κ; however, the activities of these compounds are higher against mouse pol ι than pol κ with an IC_50_ against pol ι of 19.8 µM and 32.5 µM for penicilliols A and B, respectively [34]. Collectively, these compounds have low potency as well as poor selectivity against pol κ, since they inhibit many other DNA polymerases with similar potency as they inhibit pol κ. Additionally, although penta-1,2,3,4,6-*O*-galloyl-beta-D-glucose exhibits nanomolar potency against pol κ (IC_50_ of 30 nM), it is more potent against pol α (IC_50_ of 13 nM) [35]. Thus, the value of this compound as pol κ inhibitors is lowered by poor selectivity. In contrast to the aforementioned compounds, 3-*O*-methylfunicone has been determined to exhibit high selectivity against pol κ. However, its low potency (IC_50_ of 12.5 µM) limits its utilization as a pharmaceutical or as a tool compound to probe pol κ biology [24]. Collectively, these studies emphasize the importance of the identification of pol κ inhibitors with improved potency.

Here we reported the first study to utilize the combination of qHTS and a series of secondary validation assays, including primer extension assays on non-damaged templates, replication bypass assays using site-specifically modified oligodeoxynucleotides, cell survival assays, and DNA intercalation assays for the discovery of small molecule inhibitors of pol κ. A total of 60 compounds identified through qHTS were selected as proof-of-principal chemicals and validated in radioactive primer extension assays with electrophoretic separation. The majority of these hits inhibited pol κ activity at the top concentration tested (80 µM), demonstrating the sensitivity of the qHTS assay to accurately identify positive hits and the reliability of the primer extension assays using non-damaged DNAs to confirm the hits.

After elimination of weak inhibitors, compounds with potentially reactive functionalities or other undesirable chemical features, as well as compounds for which there were no convenient commercial sources, further validation of the remaining three compounds, candesartan cilexetil, manoalide, and MK-886 were conducted by radioactive gel-based primer extension assays. The results revealed that these compounds were capable of inhibiting the ability of pol κ to catalyze synthesis on either a control non-damaged DNA template or a template adducted with the acrolein-derived ring-opened reduced form of γ-HOPdG in a dose-dependent manner with similar potency. Since the predominant role of pol κ is in DNA lesion bypass, these results demonstrated that the primer extension assays using damage-containing DNAs can effectively measure the ability of the compounds to inhibit a biologically relevant activity of pol κ.

In order to assess the ability of these compounds to target intracellular pol κ, cell survival assays were carried out by exposing cells to the combination of pol κ inhibitors and UV. The results showed that candesartan cilexetil could potentiate cellular toxicity induced by UV in XP-V cells. It cannot be ruled out that the cellular effect of candesartan cilexetil may be partly due to its effect on other proteins in addition to pol κ, including pol η and pol ι, since the compound also inhibited the activities of these polymerases *in vitro* (Table 1); however, our *in vitro* results clearly show that pol κ is inhibited by this compound. Additionally, it has been shown that the depletion of either pol η or pol ι in XP-V cells did not enhance UV cytotoxicity [16]. Collectively, these observations suggest that pol κ is inhibited by this compound in the cells, and thus validate the usefulness of this cell-based assay in identifying compounds with potential to inhibit intracellular pol κ.

Although manoalide and MK-886 could inhibit pol κ activity *in vitro*, these compounds were unable to enhance UV-induced toxicity in XP-V cells under the conditions tested. Both manoalide and MK-886 have anti-inflammatory activity; manoalide is well-known as a non-specific phospholipase A_2_ antagonist [36]–[38], and MK-886 inhibits leukotriene synthesis by blocking 5-lipoxygenase-activating protein [39]. The reason for the inability of these compounds to potentiate UV cytotoxicity could be due to their significantly lower binding affinity to intracellular pol κ relative to other cellular targets. Alternatively, these compounds may take a long time to enter the cells and bind to pol κ. Moreover, it is possible that only a small fraction of intracellular pol κ is inhibited by these compounds and the remaining pol κ may be sufficient to process UV-induced DNA lesions, resulting in unaltered cellular sensitivity to UV. Given the presence of multiple back-up TLS polymerases, nearly-complete inhibition of the activity of all intracellular pol κ may be essential for cells to present an apparent phenotype. Further understanding of the inability of these compounds to target intracellular pol κ could involve structure-activity relationship analyses. In fact, several structural analogues of these compounds exist such as secomanoalide and luffariellolide for manoalide [36] and L538,916 for MK-886 [40], thus enabling the initiation of such studies.

In summary, we presented herein the development of new strategies for the discovery of small molecules that could inhibit pol κ activity both *in vitro* and *in vivo*. The identification of chemotypes with established drug properties targeting pol κ validates this qHTS platform, as well as the secondary assays and sets the stage for exploration of significantly larger diverse collections to discover compounds with high potency and specificity towards pol κ and thus could potentially be used as pharmaceuticals. Therefore, these studies would move the research effort one step closer to the development of pol κ-targeted novel combination cancer therapeutics.

## Supporting Information

Figure S1 **Effect of candesartan cilexetil on UV-induced cytotoxicity using CellTiter-Glo Luminescent Cell Viability Assay.** XP30RO cells were treated with 24 µM of candesartan cilexetil alone, UV alone at 1.5 J/m^2^, 4.5 J/m^2^, or 7.5 J/m^2^, or in combination. Percent survival was calculated by normalizing the data using the cell survival of untreated cells. I: candesartan cilexetil.
(TIF)

Table S1 **Summary of results of qHTS and radioactive gel-based primer extension assays^1^.** ^1^In both qHTS and the radioactive gel-based primer extension assays, values of % inhibition that are over 100% and below 0% are presented as 100% and 0%, respectively. ND: not determined.
(DOC)
