# Supplementary figures and images for: A Comprehensive Strategy to Discover Inhibitors of the Translesion Synthesis DNA Polymerase κ
Source: PLoS One. 2012 Oct 8;7(10):e45032. doi: 10.1371/journal.pone.0045032 (PMC3466269; doi:10.1371/journal.pone.0045032)

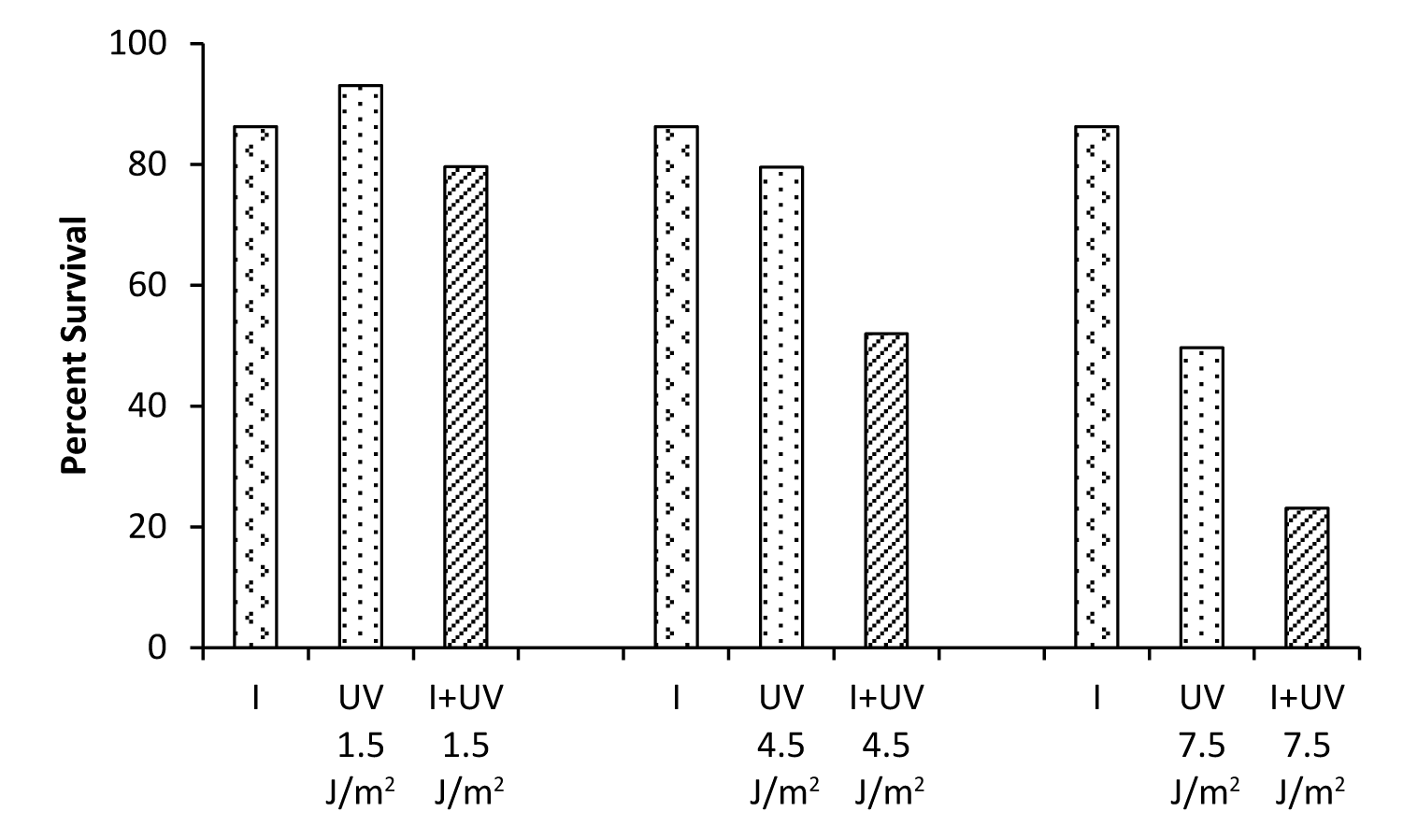

Supplement: Figure S1 — Effect of candesartan cilexetil on UV-induced cytotoxicity using CellTiter-Glo Luminescent Cell Viability Assay. XP30RO cells were treated with 24 µM of candesartan cilexetil alone, UV alone at 1.5 J/m2, 4.5 J/m2, or 7.5 J/m2, or in combination. Percent survival was calculated by normalizing the data using the cell survival of untreated cells. I: candesartan cilexetil. (TIF) [file pone.0045032.s001.tif]

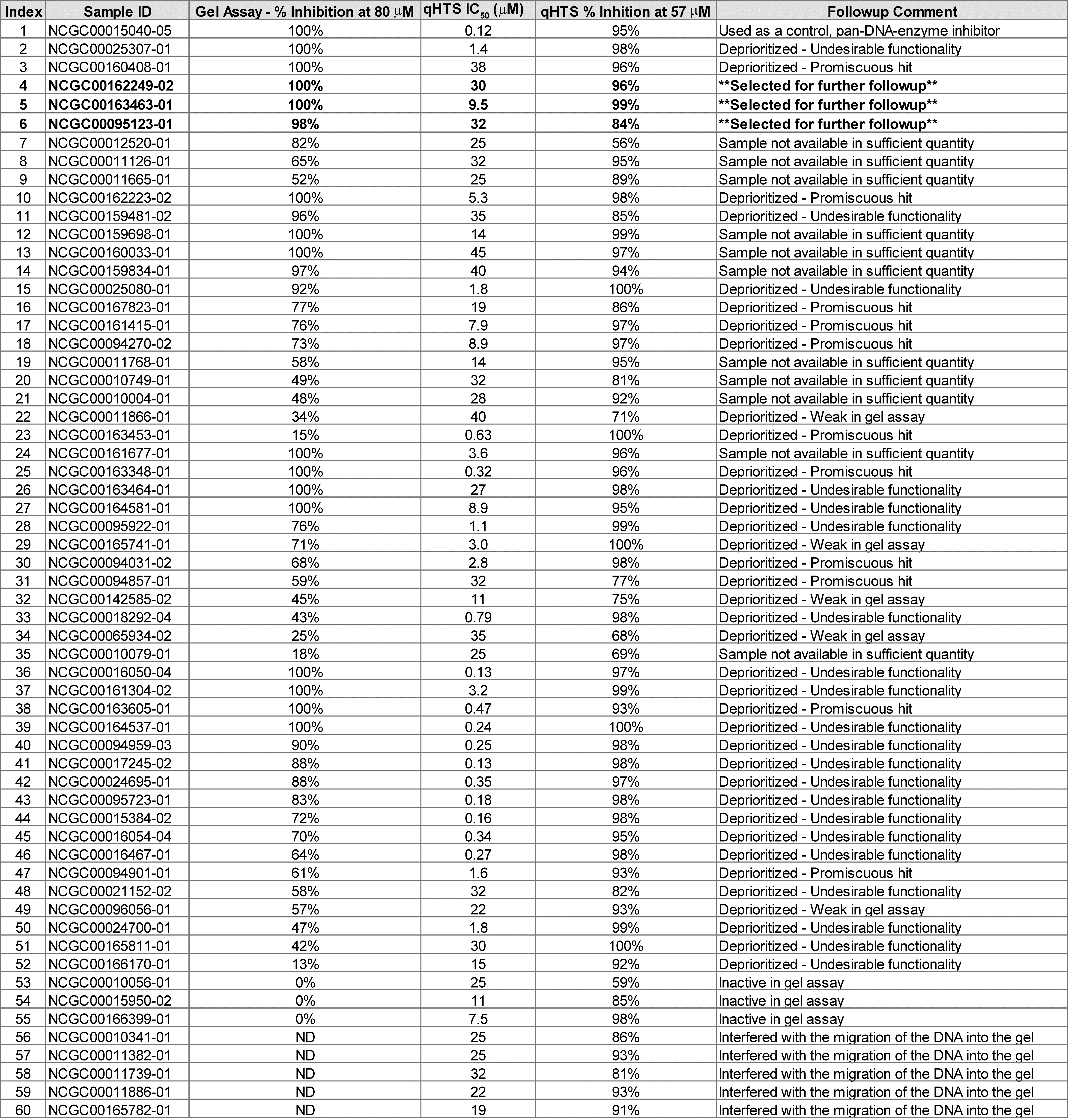

Supplement: Table S1 — Summary of results of qHTS and radioactive gel-based primer extension assays1. 1In both qHTS and the radioactive gel-based primer extension assays, values of % inhibition that are over 100% and below 0% are presented as 100% and 0%, respectively. ND: not determined. (DOC) [file pone.0045032.s002.doc]
